# Features of Sizing and Enumeration of Silica and Polystyrene Nanoparticles by Nanoparticle Tracking Analysis (NTA)

**DOI:** 10.3390/s20226611

**Published:** 2020-11-19

**Authors:** Zohair Usfoor, Katharina Kaufmann, Al Shahriar Hossain Rakib, Roland Hergenröder, Victoria Shpacovitch

**Affiliations:** Leibniz Institute for Analytical Sciences (ISAS), 44139 Dortmund, Germany; zohair.usfoor@isas.de (Z.U.); katharina2.kaufmann@tu-dortmund.de (K.K.); rakib.alshahriarhossain@rub.de (A.S.H.R.); roland.hergenroeder@isas.de (R.H.)

**Keywords:** nanoparticle tracking analysis, nanoparticles, scanning electron microscopy

## Abstract

Nanoparticle Tracking Analysis (NTA) allows for the simultaneous determination of both size and concentration of nanoparticles in a sample. This study investigates the accuracy of particle size and concentration measurements performed on an LM10 device. For experiments, standard nanoparticles of different sizes composed of two materials with different refractive indices were used. Particle size measurements were found to have a decent degree of accuracy. This fact was verified by the manufacturer-reported particle size—determined by transmission electron microscopy (TEM)—as well as by performed scanning electron microscopy (SEM) measurements. On the other hand, concentration measurements resulted in overestimation of the particle concentration in majority of cases. Thus, our findings confirmed the accuracy of nanoparticle sizing performed by the LM10 instrument and highlighted the overestimation of particle concentration made by this device. In addition, an approach of swift correction of the results of concentration measurements received for samples is suggested in the presented study.

## 1. Introduction

Sizing and quantification of nanoparticles gained increased interest from researchers working in different fields. The size and concentration of the biological nanoparticles known as extracellular vesicles (EVs) can grant invaluable information for the identification of pathological processes at early stages of disease progression [1], as well as for monitoring of the cure success [2,3,4]. The involvement of EVs in many physiological and pathological processes makes the idea of employing EVs as biomarkers very attractive. For this purpose, information regarding EV’s size, concentration and surface biochemical profile has to be acquired. However, the task of EV sizing has proven itself to be of immense difficulty due to the nano-scale sizes of these objects. EV sizing is also complicated by the fact that the refractive indices of EVs are quite close to those of water making EVs barely discernible [5].

Recent advances in particle sizing techniques have allowed many researchers in the biomedical field and other fields to determine the size of nanoparticles with a decent degree of accuracy [6]. Examples of these techniques are: resonant mass measurement (RMM) [7], transmission electron microscopy (TEM) [8], dynamic light scattering (DLS) [5,9], scanning electron microscopy (SEM) [5,9], techniques based on surface plasmon resonance (SPR) [10,11], and nanoparticle tracking analysis (NTA) [12]. Some of these techniques (i.e., SPR and NTA) enable the measurement of sample concentration as well as particle size. Each of these techniques has its own sets of advantages and drawbacks.

The technique of nanoparticle tracking analysis helps to perform direct sizing and enumeration of nanoparticles suspended in liquid [12,13]. Advantages of NTA made it a favorable technique over other nanoparticle sizing and enumeration techniques. Among NTA advantages are: the relatively short analysis time, the ability to measure both particle size and concentration simultaneously, the simplicity of operation, and lastly the moderate cost of these instruments [14].

NTA was successfully commercialized in the year 2004 [13]. Despite the existence of a large number of works [13] that have either mentioned or discussed this technology, the accuracy of NTA in the analysis of extracellular vesicles (EVs) is still under intensive investigation [3,12,15,16]. In the very recent essential work of Bachurski and colleagues [16], the comparison between two different types of analytical instruments—which employ the NTA approach for EV analysis—is given. However, this study left an opened question on whether demonstrated measurement inaccuracies depend on the sizes and concentrations of nanoparticles used in analyzed samples. This question is addressed in our work. Moreover, it was still debated whether NTA is a one-step technique to accurately measure both the particle size and the concentration of nanoparticles in a sample [3]. It appears that a certain method of calibration or further processing of the obtained data may be necessary.

In the present work, an NTA instrument is investigated in order to determine the precision of measurements performed on polystyrene and silica nanoparticles of different sizes and in different concentrations. Among the aims of this study is to explore whether different particle concentrations as well as different particle sizes can affect the inaccuracies in sizing and quantification of nanoparticles in liquid samples. However, we also strove not simply to assess vulnerabilities of LM10 measurements, but also to suggest an approach to swiftly overcome them, at least for certain conditions. Since NTA instruments, such as the LM10 device from Malvern, are often used for characterization of biological nanoparticles, it was important for the analyzed samples to resemble optical characteristics of biological nanoparticles (EVs, for example). Therefore, silica nanoparticles were employed in this investigation due to the fact that the refractive index (RI) of silica (1.47 [17]) is rather close to the one of EVs (1.37–1.42 [18]). Polystyrene nanoparticles (RI of 1.633 [18]) were also measured alongside silica nanoparticles, since the LM10 device used in the current work was initially calibrated by polystyrene nanoparticles.

## 2. Results

In this work, silica nanoparticles of six different sizes—70, 100, 200, 300, 500 and 800 nm—and polystyrene nanoparticles of four different sizes—100, 200, 300 and 400 nm—were measured by NTA technique using the LM10 device (NanoSight Ltd., Amesbury, UK; now part of Malvern Panalytical). Samples of three different concentrations of nanoparticles were prepared to investigate the level of accuracy of the resulting measurement data associated with different sample concentrations. These concentrations were chosen to be close to or within the LM10 device linear measurement range reported by literature, which is 2 to 10 $\times \;10^{8}$ particle/mL [3]. The following sections provide a preview of the obtained results.

### 2.1. Particle Size Measurement

**Figure 1:**
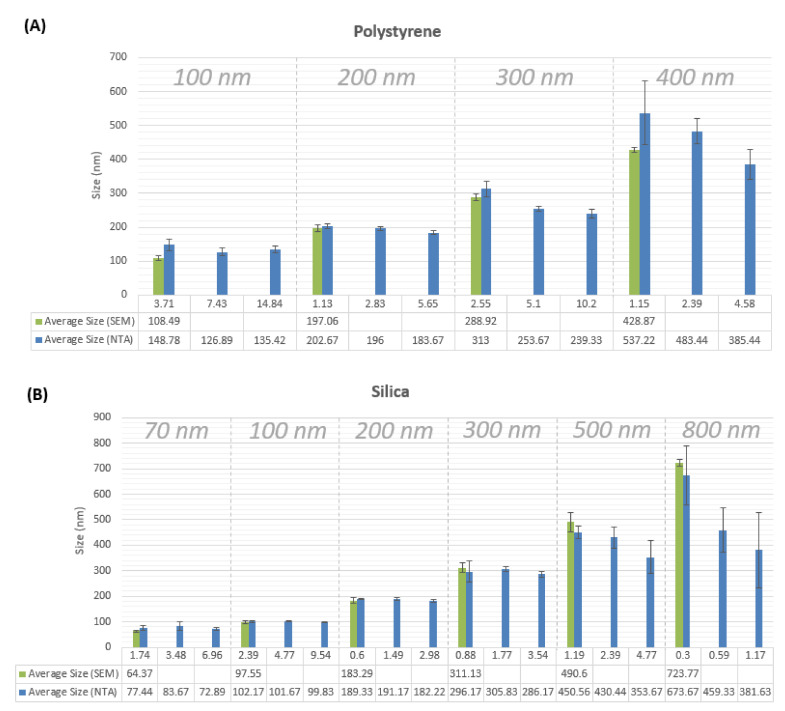
Particle size measurements by SEM and NTA of three different concentrations. The calculated values of prepared concentrations $\times 10^{8}$ particle/mL for each nominal particle size are shown on the *x*-axis. Each sample of nanoparticles is measured by NTA in triplicate by three different researchers on three separate experiments, leading to nine measurements for each sample. For SEM particle sizes, measurements were taken from images of twenty five particles for each nominal particle size. (**A**) refers to polystyrene nanoparticles, (**B**) refers to silica nanoparticles.

**Figure 2:**
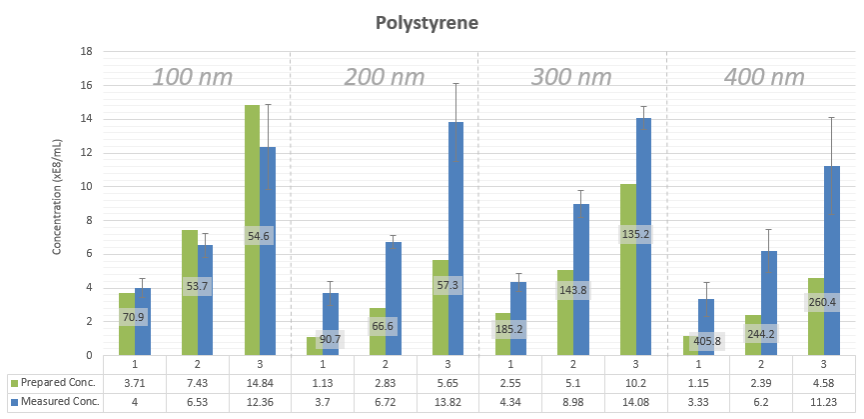
Concentration measurements of three prepared concentrations for different particle sizes for polystyrene nanoparticles as measured by NTA. Each sample of nanoparticles is measured in triplicate by three different researchers on three separate occasions, adding up to nine measurements for each sample. FWHM values for sample histograms are shown in nm in data labels.

**Figure 3:**
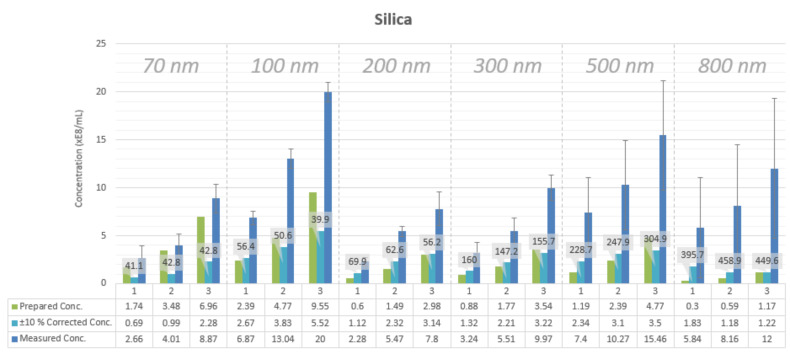
Concentration measurements of three prepared concentrations for different particle sizes for silica nanoparticles as measured by NTA. Each sample of nanoparticles is measured in triplicate by three different researchers on three separate occasions, adding up to nine measurements for each sample. The ±10% corrected concentrations represent a cut percentage of the measurement histograms. More on this can be found in Section 2.3. FWHM values for sample histograms are shown in nm in data labels.

**Figure A1:**
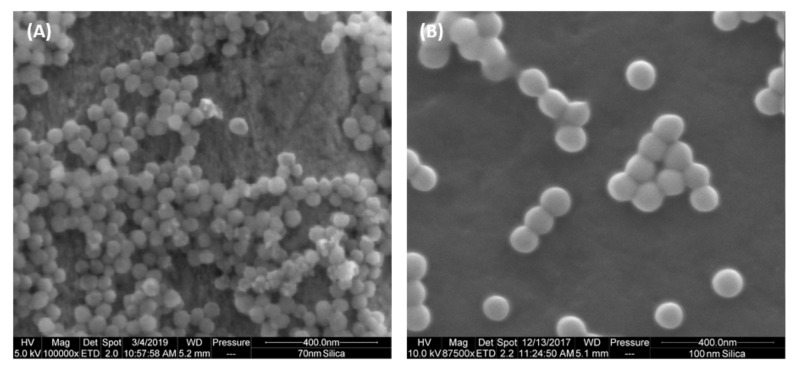

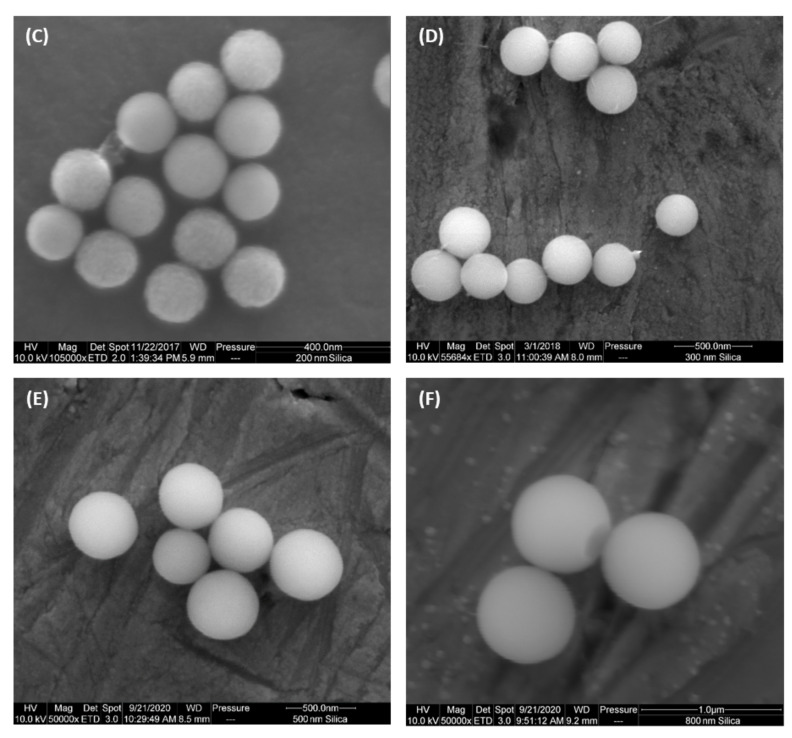
SEM images of the measured silica nanoparticle samples of different sizes. (**A**) 70 nm silica nanoparticles, (**B**) 100 nm silica nanoparticles, (**C**) 200 nm silica nanoparticles, (**D**) 300 nm silica nanoparticles, (**E**) 500 nm silica nanoparticles, and (**F**) 800 nm silica nanoparticles.

**Figure A2:**
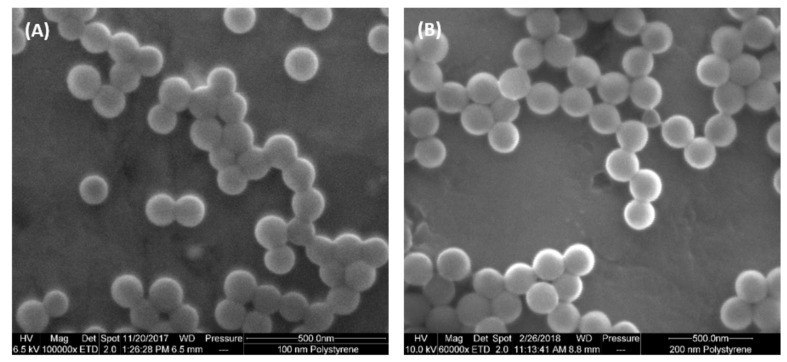

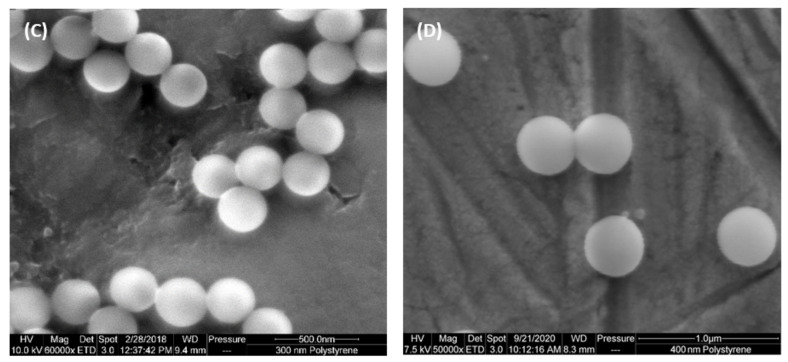
SEM images of the measured polystyrene nanoparticle samples of different sizes. (**A**) 100 nm polystyrene nanoparticles, (**B**) 200 nm polystyrene nanoparticles, (**C**) 300 nm polystyrene nanoparticles, and (**D**) 400 nm polystyrene nanoparticles.

Measured particle sizes by the NTA LM10 instrument are found to be close to those acquired by SEM measurements (Figure 1), as well as to the nanoparticle sizes reported by the manufacturer. The average percentage of deviation from the average SEM-measured particle size is about 6%. SEM images are provided in Appendix A for silica nanoaprticle samples (Figure A1) and for polystyrene nanoaprticle samples (Figure A2). For the 300 nm polystyrene n.p. sample, the measured particle size was underestimated by about 50 nm (or approximately 16% of the average size). Particle size underestimation in NTA is associated with increased noise resulting from scattering of light from bigger sizes of nanoparticles equal to or larger than 300 nm. This increased scattering is detected by the LM10 software as additional smaller particles, depreciating the overall average size of detected particles. Another reason for particle size underestimation is thought to be the high polydispersity of samples measuring 300 nm or larger in particle size, as the full width at half maximum (FWHM) of the histogram peak of these samples is higher than 100 nm (Figure 2 and Figure 3). In Figure 1, prepared concentration values are shown to demonstrate the effect of sample concentration on the particle size measurement. It is seen that generally the particle size measurement is increasingly underestimated as the sample concentration increases. This effect grows bigger with increasing the measured particle size for both materials: silica and polystyrene.

Despite the tendency for the results of particle size measurements using SEM to be underestimated, results of SEM measurements here were seen to be quite close to those reported by the nanoparticle suppliers. SEM measurements are close to those measured by NTA as well (Figure 1).

### 2.2. Concentration Measurement

Concentration of nanoparticles inside 1 mL is calculated for all samples based on the density of the nanoparticle material and the mass of nanoparticles inside 1 mL of the original nanoparticle solution. These information are provided by the nanoparticle supplier. For particles provided by Invitrogen as well as Sigma-Aldrich, the concentrations of nanoparticles are already provided by the manufacturer. The calculated values of sample concentration for these particles are found to agree with the manufacturer-provided values for sample concentration. The information provided by manufacturers about the mass of nanoparticles per 1 mL were also checked for accuracy. The mass of nanoparticles per 1 mL for 200 nm silica and polystyrene samples was measured. The mass measurement results were found to be similar to the mass values provided by the manufacturer. Therefore, it is safe to assume that our calculation method—and consequently, calculated values for nanoparticle concentrations—are accurate as well.

NTA-measured concentration values for the nanoparticle samples are found to be higher than the calculated concentration values of the prepared samples (Figure 2 and Figure 3). The measured concentration values are about 2.5 to 3 times higher than the prepared concentration values independently of particle size and particle material. An exception is 100 nm polystyrene nanoparticle samples. These samples have a minimal deviation between the measured and the theoretical concentrations, reaching 0.08% for the sample with the lowest particle concentration. This situation may occur due to the fact that the LM10 device was calibrated using 100 nm polystyrene nanoparticles.

### 2.3. Measurement of Mixtures Containing Two Particle Sizes

**Figure 4:**
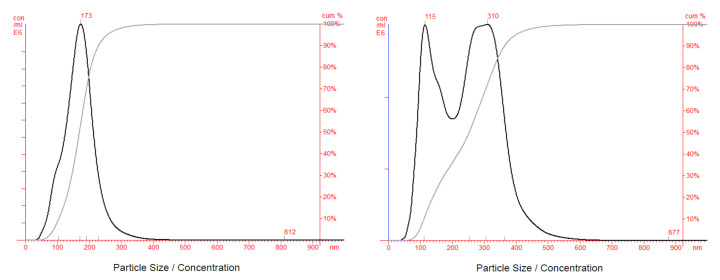
NTA histograms for mixtures of equal concentrations of two particle sizes. Left: 100 and 200 nm silica nanoparticles. Right: 100 and 300 nm silica nanoparticles. For the mixture of 100 and 200 nm particles, prepared concentration values are: for 100 nm particles: 2.98 $\times \;10^{8}$ particle/mL, for 200 nm particles: 2.99 $\times \;10^{8}$ particle/mL. Total measured concentration: 13.42 $\times \;10^{8}$ particle/mL. ±10% corrected concentration for the 200 nm peak: 4.35 $\times \;10^{8}$ particle/mL. For the 100 nm particles, a noticeable peak could not be found in the size distribution that allows for the application of our correction method.

Mixtures of equal concentrations of 100 and 300 nm silica nanoparticles as well as mixtures of equal concentrations of 100 and 200 nm silica nanoparticles are measured by NTA to test the performance of measurements for polydisperse samples. It is found that for the mixture of 100 and 200 nm particles, the measured particle concentration for the 100 nm particles is greatly underestimated in comparison with the concentration for the 200 nm particles (Figure 4, left). However, measured concentrations for the 100 and 300 nm particle mixture were equally overestimated (Figure 4, right). This suggests that, for a polydisperse sample of two sizes of nanoparticles close in refractive index to that of silica, particles must be of a size difference equal to about twice the size of the smaller particle group in order for the concentrations of the two particle groups to be equally estimated. One solution to this problem can be to centrifuge the nanoparticle sample with density gradient media to separate different particle sizes, and then to measure each particle size group separately.

**Figure 5:**
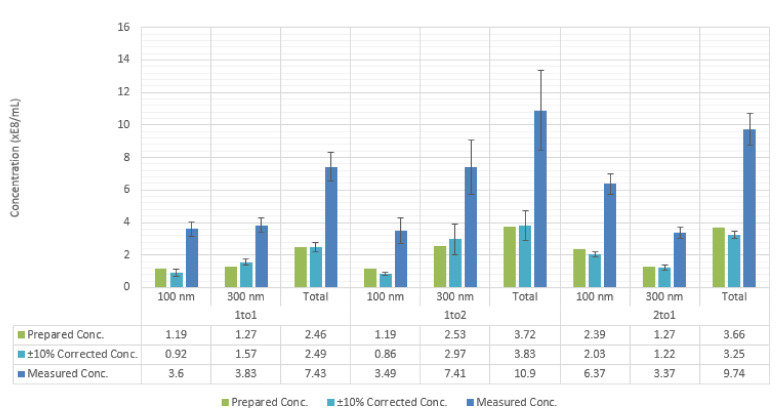
NTA concentration measurements for mixtures of 100 and 300 nm silica nanoparticles, composed of three different ratios of 100 to 300 nm particles. The two particle size peaks are separated from the measurement histogram then corrected separately using the correction method mentioned in this article.

The method of correction of the measured concentration mentioned in a following section was tested here as well. It was found that this method is still valid when applied to separate size peaks for a mixture of 100 and 300 nm silica nanoparticles. It is valid for correcting the total solution concentration as well (Figure 5).

### 2.4. A Swift Approach to Correct the Results of NTA Concentration Measurements for Samples Containing Silica Particles

Since measured sample concentrations are demonstrated to be higher than the prepared nanoparticle concentrations, a swift correction of the concentration values, which are received as a result of NTA measurements, is desirable. Such a swift correction method is especially important for biological samples containing any type of biological nanoparticles, such as viruses or extracellular vesicles. From the optical characteristics (refractive indices), silica nanoparticles are closer to biological nanoparticles than polystyrene nanoparticles. Thus, we aimed to suggest a correction approach to process NTA concentration measurement results for samples containing silica nanoparticles.

The measurement data of one sample—i.e., the histogram—contains measured concentrations for each particle size from 1 to 2000 nm. Therefore, the suggested correction is to consider for concentration measurement results only measured concentrations, which fall within ±10% of the measured mode particle size. For example, if the measured mode particle size is 100 nm, then only measured concentrations of sizes from 90 to 110 nm are considered—i.e., summed together. The ±10% corrected concentration values are closer to the prepared concentration values for silica nanoparticles of diameters more than 100 nm (Figure 3). Moreover, the same swift correction approach is applicable to separate peaks in mixtures of particles as demonstrated earlier (Figure 5).

**Figure 6:**
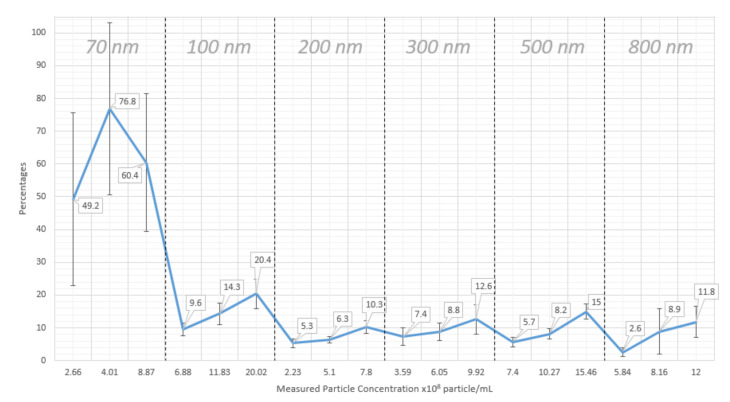
Correction percentages to match prepared concentrations for different silica nanoparticle sizes and for three different concentrations with correction percentages in data labels for each point. Each point in this graph is the average correction percentage for nine separate measurements of the same sample.

We also would like to describe how the presumed ±10% correction value for concentration measurements was validated. For each measurement, the correction percentage of the histogram that is required to match the prepared concentration value was looked for. For a file of one measurement that contains all measured concentrations for sizes from 1 to 2000 nm, correction percentages on the concentration values from ±1% to ±100% were tested. If one of the tested correction percentages is found to result in a concentration, that is close to the prepared concentration for the sample of this measurement file, this percentage is noted down. For concentration measurements of silica nanoparticles, it is found that a correction percentage of ±10% approximates most of the prepared concentration values (Figure 6).

### 2.5. Influence of Camera Level Changes on Accuracy of Concentration

A built-in feature of the LM10 software is the ability to change the brightness and contrast of the video recording of particles as they move under Brownian motion. This is done by changing the camera shutter speed and gain. The automatic recommended settings of the proprietary LM10 software provide preset levels of the camera shutter speed and gain labeled as “camera levels”. It was tested whether the change in the recommended camera levels would affect the measured particle concentration by NTA.

**Figure 7:**
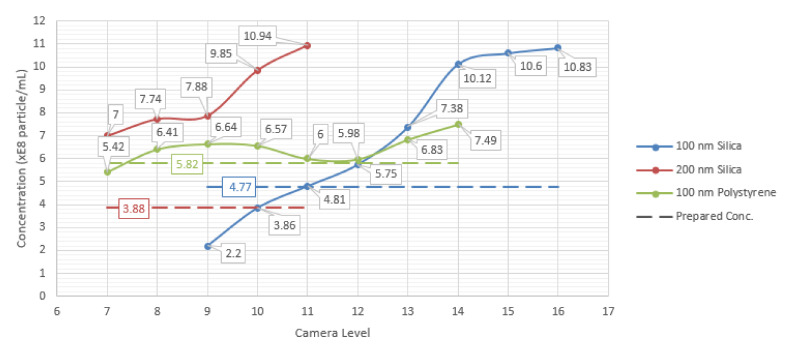
Effect of changing the camera levels on the measured concentration for 100 nm silica, 200 nm silica and 100 nm polystyrene samples. Prepared concentration values for each of the samples are shown as dotted lines. The last point in each sample line corresponds to the recommended camera level by the software for the measured sample.

Recommended camera levels are shown by the NTA software by the disappearance of a “DARK!” sign in the software interface. The results of the camera level investigation are represented in Figure 7. Here, each sample is measured three times at each of the camera levels around the recommended camera level. The averages of the three measurements for each of the camera levels are compared.

It was found that around recommended camera levels, there were no significant differences in the measured concentration values. The measured concentrations were lower in the cases where the camera levels are lower than the recommended by software (Figure 7). The reason for this is that under lower camera levels, low brightness and contrast hinders the detection of a number of particles within the camera depth of field, especially those moving in further distances away from the camera objective.

It was also noticed that for the 100 nm silica nanoparticles, recording at a camera level of 11 has led to a measured concentration that is very close to the actual prepared value. Measurements performed at this camera level were continuously accompanied by sign “DARK” indicating that they were performed under non-optimal conditions. Thus, being forced to measure particles under non-optimal conditions, the instrument may provide concentration values rather close to calculated prepared concentration. Thus, one may jump to hasty conclusions that for each particle size measured by the LM10 device, there could be a certain camera level for which the concentration measurement would be rather accurate. However, this is not the truth and this fact is demonstrated for the 200 nm silica sample, for example, where the measured concentration does not reach the value of the prepared concentration even near the lowest level of the preset camera levels. Nevertheless, it is seen that the recommended camera levels can be used, but with additional processing of the resulted data, as discussed in the previous section.

## 3. Discussion

In spite of the fact that inaccuracies in the NTA measurements of non-biological particles as well as EVs have been previously reported in several independent studies [3,8,12,15,16], it remained unclear whether differences in materials, sizes and concentrations of particles in analyzed samples could affect demonstrated tendencies. Thus, it was important to analyze samples containing particles that vary in materials and sizes, and can be prepared in different concentrations. Moreover, we aimed to investigate at least one type of particle materials (silica), which resembles the optical characteristics of biological particles in terms of refractive indices. Samples of 70, 100, 200, 300, 500 and 800 nm silica nanoparticles and 100, 200, 300, and 400 nm polystyrene nanoparticles were measured by the LM10 instrument (Malvern), which utilizes principles of NTA. Each particle size was measured in three different concentrations, which were decided according to the previously reported most accurate measurement range of this device [13]. Two parameters were investigated in this work: the size of nanoparticles and their concentration in analyzed samples. The aim of this study, was to assess whether the inaccuracies of the LM10 NTA measurements can be affected by a variation in concentrations or sizes of nanoparticles in the analyzed samples. SEM measurements performed in this work helped to validate particle size measurements carried out by the LM10 NTA instrument.

We demonstrated that particle size measurements for particles ranging in size from 70 nm to 300 nm are accurate, deviating only from ±2% to a maximum of ±7% of size values measured using SEM images of the nanoparticle samples. For larger particle sizes, measurements tend to be underestimated for concentrations higher than 4 $\times \;10^{8}$ particle/mL.

On the other hand, measured concentration values are consistently higher than the calculated values of the prepared concentrations. Increments of about 2.5 times the prepared concentration values are present. These findings are in agreement with previously reported inaccuracies for NTA instruments [16]. The swift correction approach used in our work aided in reducing the inaccuracies between measured concentration values of silica nanoparticles and actual prepared values of particle concentrations in monodisperse samples as well as individual particle size groups in polydisperse samples.

## 4. Materials and Methods

The following is a description of the various experimental procedures carried out throughout this work, including the calculation of concentrations as well as protocols followed for NTA and SEM measurements.

### 4.1. Prepared Concentration Calculations

Alongside measured concentrations provided by the NTA device, initial calculation of the concentrations of prepared samples is carried out. The calculation of concentrations requires the knowledge of the mass of nanoparticles in 1 mL of suspension. This information is provided by the manufacturers of n.p. in our case. However, we measured it independently in order to confirm its accuracy. For this purpose, 200 nm silica and polystyrene particle samples were taken as examples. Then, 2 mL of each sample were dried and the mass of the nanoparticles alone was measured. For the 200 nm silica particle sample, the mass measured by us was identical to the one reported by the manufacturer. For the 200 nm polystyrene particle sample, there was a difference of 0.0015 g for 1 mL of suspension. These results confirmed that information about nanoparticle masses provided by the manufacturers can be safely used for calculations.

In order to calculate the number of nanoparticles in 1 mL of nanoparticle suspension, we divide the volume of nanoparticles inside a 1 mL volume of nanoparticle suspension by the volume of a single nanoparticle. To do so, a few parameters should be known: density of the nanoparticle material (i.e., of silica and polystyrene), mass of nanoparticles in 1 mL of stock solution (as provided by the nanoparticle supplier), and the actual nanoparticle size (can also be provided by the supplier).

Since $\rho =\frac{m}{V}$, the volume of 1 mL of suspension can be calculated as:

(1) $$V_{in1mL}=\frac{m}{\rho }$$

The volume of one spherical nanoparticle is:

(2) $$V_{of1n.p.}=\frac{4}{3}\pi r^{3}$$

Then the concentration of nanoparticles in a sample would be:

(3) $$Concentration=\frac{V_{in1mL}}{V_{of1n.p.}}$$

The final formula then can be simplified as:

(4) $$Concentration=\frac{3m*10^{-6}}{4\rho \pi {\left(r*10^{-9}\right)}^{3}}÷dilutionfactor$$

### 4.2. Nanoparticle Sample Preparation

Nanoparticle standards for silica and polystyrene were supplied by Kisker-biotech, Germany for the 70 (order number: PSI-0.07COOH), 200 (order number: PSI-0.2COOH), 300 (order number: PSI-0.3COOH), 500 (order number: PSI-0.5COOH) and 800 nm silica nanoparticles (order number: PSI-0.8COOH), and 100 (order number: PPs-0.1COOH) and 200 nm polystyrene nanoparticles (order number: PPs-0.2COOH). The 100 nm silica nanoparticles (order number: 803308) were supplied by Sigma-Aldrich, Germany. The 300 (order number: S37492) and 400 nm polystyrene nanoparticles (order number: S37493) were supplied by Invitrogen, Germany.

To measure samples of nanoparticle suspensions by NTA or SEM, diluted suspensions were prepared from the highly concentrated stock suspensions.

The solvent used in the preparation of different dilutions was filtered distilled water. To prepare it, first, distilled water was generated by a water distillation device (produced by Millipore, Germany; now rebranded as Merck). After that, the distilled water was filtrated using a peristaltic pump through two filters: (a) 0.45 μm pore size filter (produced by Whatman, UK) and (b) 0.025 μm pore size filter (by Millipore, Germany; now rebranded as Merck).

The procedure followed for preparing a sample of a nanoparticle suspension started with taking an aliquot of a maximum of 1 mL of the original nanoparticle suspension. Before aliquoting, the original suspension was subjected to an ultrasonic bath for 20 min to separate any nanoparticle agglomerates. Aliquoting was carried out inside a clean air enclosure (also called: glove box) filled with argon gas to prevent contamination of the original suspension or the aliquot by dust or airborne bacteria. The aliquots were kept afterwards in a 4 ${}^{{}^{\circ}}$C refrigerator.

Since nanoparticle aliquots contain concentrations in the order of $10^{12}$ to $10^{14}$ particles/mL, diluted suspensions of 1:100 in filtered water were prepared from the aliquot, which served as the source for further dilutions.

### 4.3. NTA Measurement Protocol

To carry out NTA measurements, an LM10 device (produced by NanoSight, Amesbury, UK; now part of Malvern Panalytical), equipped with a CCD camera and a 405-nm laser, was utilized.

Before each NTA measurement session, the filtrated water used for sample dilution, was checked for the presence of nanoparticles to serve as a negative control. In many cases, the negative control measurement detected no particles at all. In some cases, however, the measured negative control was less than 0.13 $\times \;10^{8}$ particle/mL, and of less than 100 nm in size, which was deemed to be acceptable. This is due to the fact that this comprised a very small percentage of less than 5% of the measured concentrations of the least concentrated samples.

Directly before a sample was analyzed by the LM10 instrument, the sample was subjected to an ultrasonic bath for 10 min to separate any agglomerates, then mixed by a vortex mixer for 10 s to homogenize the suspension. The minimum volume recommended for analysis is 500 μL. Therefore, the sample was injected afterwards by a 1 mL syringe into the LM10 analysis chamber. A video of 90 s duration of the particles under Brownian motion was recorded afterwards using the NanoSight software. Afterwards, three sets of triplicated measurements were taken on different days for each one of the nanoparticle samples.

### 4.4. SEM Measurement Protocol

In order to validate the results of particle size measurements given by NTA, images of nanoparticles were taken by a Quanta 200 F scanning electron microscope (FEI company, Hillsboro, OR, USA; now part of Thermo Fisher Scientific Inc., Waltham, MA, USA) then analyzed by ImageJ software (developed by Wayne Rasband, in the National Institutes of Health (NIH), Bethesda, MD, USA). For SEM analysis, 2 mL nanoparticle suspensions were prepared, then drops of 100 μL of these suspensions were deposited on aluminum and brass pins, and 20 μL on glassy carbon (also called vitreous carbon) pins that fit inside the SEM analysis chamber. There should be an adequate number of nanoparticles on the specimen pins to facilitate finding particles using SEM, therefore the nanoparticle suspensions used in this case were diluted to concentrations in the order of $10^{9}$ particles/mL. Afterwards, they were dried in a standard laboratory drying oven on approximately 80 ${}^{{}^{\circ}}$C for 30 min. The specimen pins with dried suspensions were then inserted in the SEM analysis chamber for imaging. Factors that affect the image quality are usually the focus, astigmatism value, brightness and contrast. These factors were modified until an image of acceptable clarity is acquired.

After that, analysis by ImageJ software allows for the determination of particle sizes from given images. This starts by measuring the diameter of the particle in pixels (by drawing a line on the particle by the user) then converting the pixel count into nanometers by providing the software with the image scale, which is represented as a line (drawn by the SEM software on the image) with the equivalent nanometer value shown underneath. Different types of specimen pins were used (Glassy carbon, brass, and aluminium pins) in order to test the image quality difference acquired by pins of different materials and to choose the clearest image.

## Abbreviations

The following abbreviations are used in this manuscript:

NTA	Nanoparticle Tracking Analysis
SEM	Scanning Electron Microscopy
RMM	Resonant Mass Measurement
TEM	Transmission Electron Microscopy
DLS	Dynamic Light Scattering
SPR	Surface Plasmon Resonance
N.P.	Nanoparticle
FWHM	Full Width at Half Maximum
RI	Refractive Index
EV	Extracellular Vesicle
